# Non-invasive pressure–volume loops by cardiovascular magnetic resonance and outcome in ST–elevation myocardial infarction

**DOI:** 10.1093/ehjimp/qyag055

**Published:** 2026-03-29

**Authors:** Theodor Lav, Kasper Kyhl, David Nordlund, Henning Kelbæk, Lars Køber, Dan Høfsten, Jacob Lønborg, Henrik Engblom, David Erlinge, Thomas Engstrøm, Håkan Arheden

**Affiliations:** Clinical Physiology, Department of Clinical Sciences Lund, Lund University, Skåne University Hospital, Lund 221 85, Sweden; Department of Cardiology, Odense University Hospital, Odense, Denmark; Clinical Physiology, Department of Clinical Sciences Lund, Lund University, Skåne University Hospital, Lund 221 85, Sweden; Department of Cardiology, Zealand University Hospital, Roskilde, Denmark; Department of Cardiology, Rigshospitalet and University of Copenhagen, Copenhagen, Denmark; Department of Cardiology, Copenhagen University Hospital, Rigshospitalet, Copenhagen, Denmark; Department of Cardiology, Copenhagen University Hospital, Rigshospitalet, Copenhagen, Denmark; Clinical Physiology, Department of Clinical Sciences Lund, Lund University, Skåne University Hospital, Lund 221 85, Sweden; Cardiology, Department of Clinical Sciences Lund, Lund University and Skane University Hospital, Lund, Sweden; Department of Cardiology, Copenhagen University Hospital, Rigshospitalet, Copenhagen, Denmark; Clinical Physiology, Department of Clinical Sciences Lund, Lund University, Skåne University Hospital, Lund 221 85, Sweden

**Keywords:** acute myocardial infarction, outcome, pressure–volume loops, cardiac magnetic resonance, heart failure

## Abstract

**Aims:**

We aimed to investigate if non-invasive pressure–volume (PV) loop variables by cardiovascular magnetic resonance (CMR) are associated with all-cause mortality or heart failure compared to mean arterial blood pressure (MAP), left ventricular ejection fraction (LVEF), and stroke volume (SV) in patients with revascularized ST–elevation myocardial infarction (STEMI).

**Methods and results:**

A total of 653 STEMI-patients from the DANAMI-3 trial underwent CMR and brachial blood pressure registration after revascularization. Volumetric CMR-data and brachial blood pressure were used to generate PV loops and calculate arterial elastance, contractility, energy per ejected volume, external power, potential energy, stroke work, ventricular efficiency, and ventriculoarterial coupling. The primary outcome was a composite endpoint of all-cause mortality or hospitalization for heart failure. A total of 39 patients met the primary outcome during a maximal follow-up time of 4.7 years. Potential energy (HR 1.38, 95% CI 1.02–1.88) and ventriculo-arterial coupling (HR 1.35, 95% CI 1.03–1.78) were associated with the primary outcome after adjustments for age, sex, and infarct size. LVEF (HR 0.74, 95% CI 0.49–1.10), MAP (HR 1.12, 95% CI 0.80–1.58), and SV (HR 1.02, 95% CI 0.68–1.54) did, however, not show an association with the primary outcome.

**Conclusion:**

Non-invasive pressure–volume loop variables are prognostic of all-cause mortality and hospitalizations for heart failure independent of age, sex, and infarct size and may provide incremental prognostic information to left ventricular ejection fraction and infarct size for clinical outcome after myocardial infarction. Thus, non-invasive PV-loop variables could potentially be used for early treatment guidance in post-STEMI patients.

## Graphical Abstract

**Graphical Abstract:**
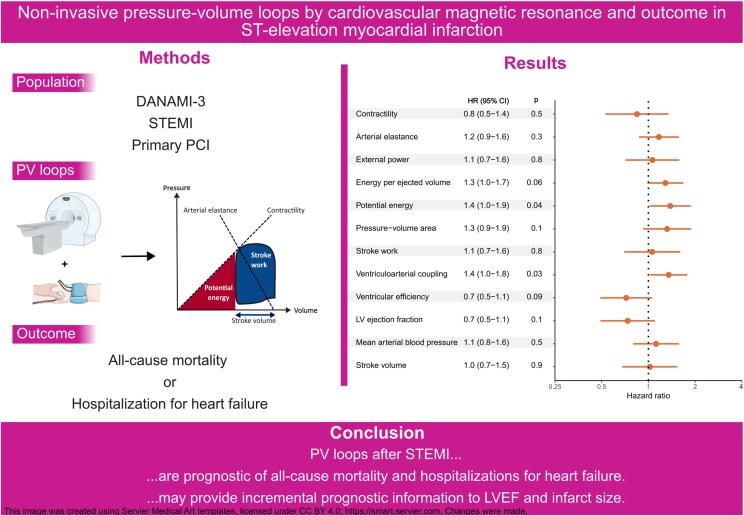

## Introduction

Clinical outcomes after myocardial infarction range from no symptoms to fatal cardiac failure.^1^ Accurate prognosis of outcomes is essential to guide clinical management and to prevent complications such as heart failure and cardiac death after acute myocardial infarction.^2^ For patients with ST–elevation myocardial infarction (STEMI) the standard treatment is acute revascularization by percutaneous coronary intervention (PCI). Post-revascularized STEMI patients are recommended to undergo a clinical risk assessment for short-term mortality, including blood pressure as a marker for future events.^2^

Echocardiography or cardiovascular magnetic resonance (CMR) has been shown to be valuable for outcome prediction in patients with myocardial infarction as well as for assessing the need for a primary preventive implantable cardioverter defibrillator (ICD).^2^ Current guidelines recommend the use of left ventricular ejection fraction (LVEF) to guide heart failure medication, and in assessing the need of an ICD.^3^ However, LVEF cannot fully predict the occurrence of hospitalization for heart failure or cardiac death.^4,5^ A more comprehensive method might be able to more precisely evaluate the risk of a variety of future events in post-STEMI patients.

Pressure–volume loops (PV-loops) combine cardiac pressures and volumes which may provide an incremental prognostic value to pressure or volume alone. However, PV-loops have previously required invasive measurements limiting its use in clinical trials. Recently, a novel non-invasive method of generating PV-loops has been developed and validated.^6–9^ The method enables assessment of PV-loop variables in large patient populations and has been applied in a range of clinical and experimental settings.^10–12^ One of these studies showed that non-invasive PV-loop variables by CMR are associated with adverse cardiac remodelling post-STEMI.^13^ Furthermore, non-invasive PV-loop variables have been shown to be associated with major adverse cardiovascular events in patients with heart failure and reduced LVEF.^14^ However, the relationship between non-invasive PV-loop variables by CMR and clinical outcomes such as mortality or heart failure has not yet been investigated.

Therefore, we aimed to investigate if non-invasive PV-loop variables are associated with all-cause mortality or heart failure compared to mean arterial blood pressure (MAP), LVEF, and stroke volume (SV) in patients with revascularized STEMI.

## Methods

This is a cohort sub-study within the DANAMI-3 randomized controlled trial.^15^ All patients were consecutively recruited at Rigshospitalet in Copenhagen, Denmark between 2011 and 2014. The manuscript was written according to STROBE guidelines. The data underlying this article will be shared on reasonable request from the corresponding author.

### Study design

In total, 653 adult patients meeting STEMI criteria with acute chest pain onset within 12 h before revascularization by primary PCI were included in the study.^15^ The diagnostic criterion for STEMI was ≥0.1 mV ST-elevation in at least two neighbouring leads or first-time left bundle branch block.^15^ A CMR examination was performed within 48 h after revascularization. Sphygmomanometric brachial blood pressure was measured in supine position during or immediately after the CMR examination. Follow-up for cardiovascular outcomes were performed by Danish national registries for at least 2 years post-revascularization and the outcomes have previously been described.^15^ A composite endpoint of all-cause mortality and hospitalization for heart failure was decided to constitute the primary endpoint and the individual components of the primary endpoint were chosen as secondary endpoints. PV-loop variables, MAP, LVEF, SV, and infarct size constituted explanatory variables. Exclusion criteria included stent thrombosis, cardiogenic shock or unconsciousness, coronary artery bypass surgery indication, percutaneous coronary intervention (PCI) not possible, previous myocardial infarction in the infarct related artery, renal dysfunction, pacemaker, chronic atrial fibrillation, coagulopathy, increased haemorrhagic tendency, medical intolerance, pregnancy and if patients were unable to give informed consent.^15^ Main exclusion criteria from CMR were claustrophobia and renal dysfunction. The study was approved by the regional ethics committee and the institutional ethics committee in Denmark. All patients provided written informed consent prior to inclusion.

### Cardiovascular magnetic resonance imaging

The CMR protocol for this study was previously described.^16,17^ Cardiovascular magnetic resonance imaging was performed on a 1.5 T scanner (Avanto or Espree, Siemens Medical Solutions, Erlangen, Germany). In short, steady-state free precession (SSFP) cine imaging of 2-, 3- and 4-chamber views was performed followed by a short axis (SAX) stack of cine images covering the left ventricle (CMR parameters: 25 frames/cardiac cycle, 8 mm slice thickness and no slice gap). A T2-weighted short tau inversion recovery (STIR) sequence was performed to evaluate the myocardium at risk (CMR parameters: 8 mm slice thickness and no slice gap). Late gadolinium enhancement (LGE) imaging was performed approximately 10 min after administration of Gadolinium contrast (Gadovist, Bayer Schering, Berlin, Germany) to allow for infarct visualization (CMR parameters: 8 mm slice thickness and no slice gap).

### Image analysis

Volumetric CMR analysis was performed in Segment v4.0 R11044 (http://segment.heiberg.se). The endocardial border was delineated in end-diastole, end-systole, and diastasis for each MR scan. The papillary muscles were included in the blood pool. An interpolation of the endocardial delineation was performed between end-diastole, end-systole, and diastasis for all time frames according to a previously validated method.^11^ Basal and apical adjustments were performed when necessary.^10^ In cases with one missing midventricular or apical SAX slice, an interpolation between the adjacent SAX slices was performed in end-diastole, end-systole, and diastasis. An apical slice was delineated by adding an apical slice and delineating in comparison to the 2-, 3- and 4-chamber cine images. Delineation of myocardium at risk was performed by the 2 SD from the remote method, and infarct size by the 5 SD from the remote method, as part of the original publication using CVI42 (Circle Cardiovascular Imaging Inc, Calgary, Canada).^16^

### Pressure–volume loop variables and conventional measurements

**Figure 1:**
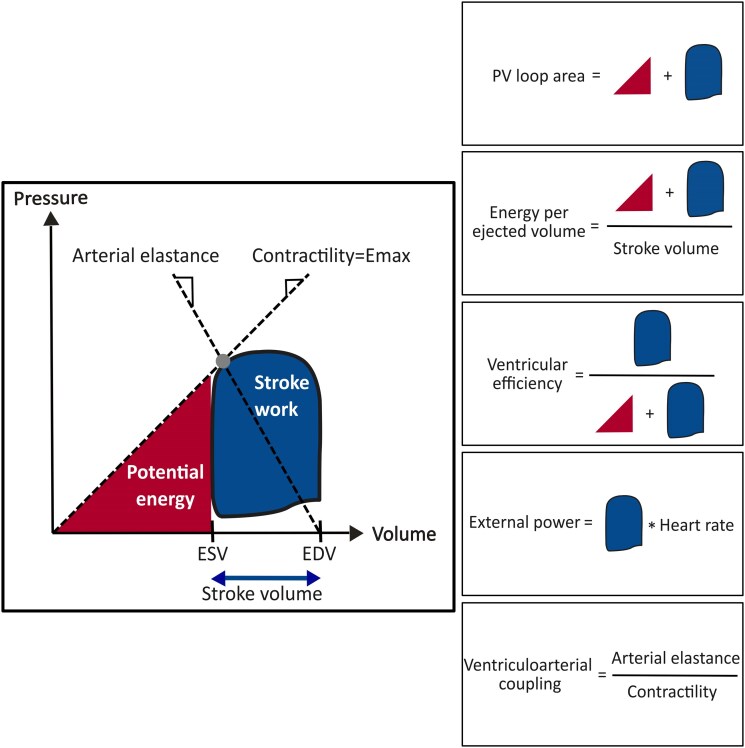
The pressure–volume loop and its derived variables. The grey dot represents the point of maximal elastance (*E*_max_). Contractility and arterial elastance were defined as the slope of the lines between *E*_max_ and origo, and *E*_max_ and end-diastolic volume (EDV). Stroke work was defined as the blue area of the PV loop. Potential energy was defined as the red area under the contractility line between origo and the PV loop. ESV, end-systolic volume.

Pressure–volume loops were generated using a previously validated non-invasive algorithm.^6,8^ The method uses volumetric CMR data from one cardiac cycle together with sphygmomanometric brachial blood pressure as input to generate a PV-loop.^6^ The left ventricular pressure was derived from a time-varying elastance model which was scaled in peak-systole by the brachial blood pressure and in end-diastole by an assumed pressure of 5 mmHg for subjects with CMR-derived LVEF ≥ 40% and to 12.5 mmHg for LVEF < 40%.^6^ The following variables were calculated from the PV-loop and are presented in *Figure 1*: Contractility represents the left ventricular contractile ability independent of loading conditions and was calculated by the slope of the line between origo and the maximal elastance. Arterial elastance represents the stiffness of the arterial compartment and was calculated by the slope of the line between the maximal elastance and the end-diastolic volume point on the *x*-axis. Stroke work represents the ventricular mechanical work used for ejecting blood and was defined as the area of the PV-loop. Potential energy represents the energy needed to generate the pressure-buildup for the ventricle to start ejecting blood and was defined as the area restricted by the contractility line, the *x*-axis and the PV-loop. These variables were used to calculate PV area (=stroke work + potential energy), ventriculoarterial coupling (=arterial elastance/contractility), external power (=stroke work * heart rate), ventricular efficiency (=stroke work/PV area), and energy per ejected volume (=PV area/stroke volume). Conventional measurements were MAP, SV, and LVEF. The formulas for PV loop variables and conventional measurements are presented in Supplementary data online, *Table S1*.

### Clinical outcomes

The primary outcome was defined as a composite endpoint of all-cause mortality or hospitalization for heart failure. DANAMI-3 also includes myocardial infarction- and revascularization endpoints in the DEFER- and PRIMULTI RCT’s.^15^ These outcomes were deemed non-relevant, however, for the prognostic value of post-infarct cardiac physiological properties as assessed by PV loops. The primary outcome in our study is agreeing with the DANAMI-3 iPOST study, which calculated to find a 25% relative reduction in outcome by including 1100 patients in that study.^15^ Our study, with above half the sample size of DANAMI iPOST, could therefore not detect the same relative reduction in outcome with the same power. Secondary outcomes were defined as the individual endpoints of the composite primary outcome.

### Statistical analysis

Numerical data were presented as mean ± standard deviation (SD) or median [interquartile range] for continuous variables. Categorical variables were presented by absolute value and percentage of population. Normal distribution of data was controlled by QQ-plots. The logarithm of a variable was calculated when appropriate to fit a normal distribution. A correlation matrix using Spearman’s rho was made after assessing scatterplots of the associations between covariates and predictors. Cumulative incidences at maximal follow-up were assessed for the primary outcome, all-cause mortality, and heart failure. Unadjusted- and adjusted cox regression models were applied for the primary outcome and all-cause mortality, respectively. Fine and Gray regression models were applied for heart failure as outcome to account for all-cause mortality as competing risk. Age, sex, and infarct size were used as covariates to adjust for their associations with PV-loop variables, and the primary outcome.^10,13,18–22^ The same covariates together with history of diabetes was applied in a second model, as the latter has been shown to be associated with ventricular remodelling and heart failure development.^23,24^ The secondary outcome variables were only adjusted for infarct size due to low number of events. Hazard ratios were expressed per standard deviation from the mean to make different variable units comparable. The proportionality assumption was controlled by Shoenfield's residuals. A Spearman correlation of <0.8 was tested to avoid collinearity between each PV-loop or conventional variable and each continuous covariate. A concordance index (C-index) was calculated for each adjusted and unadjusted model to compare the discriminative ability between the different models. The confidence level was set to 95% and *P*-values <0.05 indicated statistical significance. All statistical analyses and figures were performed using R V4.2.2 (www.R-project.org).

## Results

**Figure 2:**
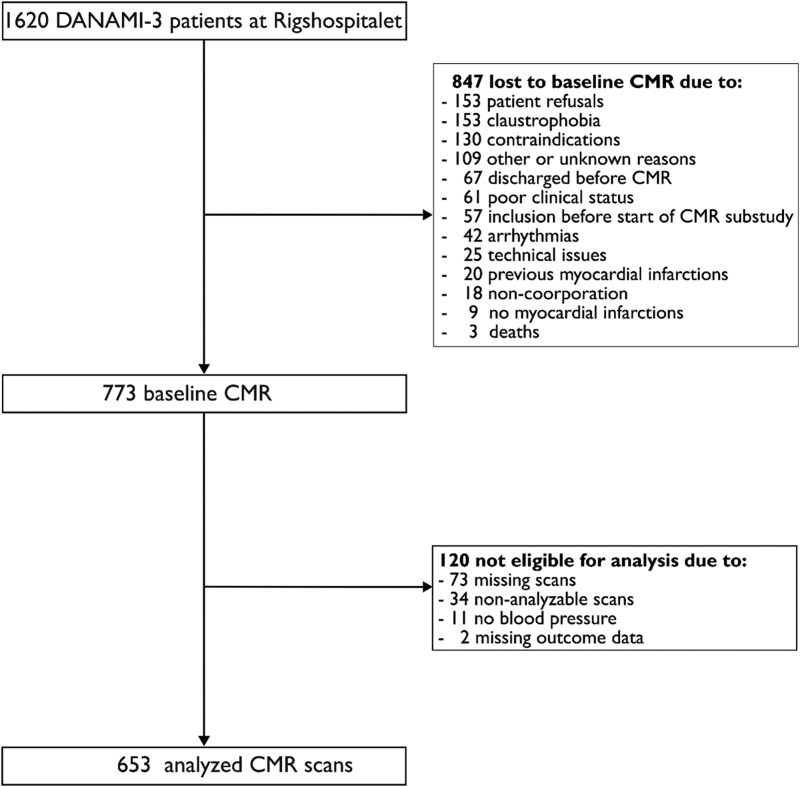
Patient flow chart. CMR, cardiovascular magnetic resonance.

A total of 653 STEMI patients with an acute CMR in the DANAMI-3 trial were included and analysed in this study. A study flow chart is shown in *Figure 2*.

### Population characteristics

Patient characteristics are shown in *Table 1*. Median symptom duration from symptom onset to percutaneous coronary intervention was 170 min (IQR: 126–271). Left anterior descending artery was the culprit vessel in 38% of the patients. Mean myocardium at risk, median infarct size, and mean (SD) LVEF by CMR were 33 (11) %, 16% (IQR: 8–25%), and 49 (10) %, respectively. Median and interquartile range of PV-loop variables and conventional measurements are shown in Supplementary data online, *Table S1*. A correlation matrix between PV loop variables and conventional measurements are shown in Supplementary data online, *Figure S1*. Non-included patients’ characteristics compared to included patients’ characteristics are shown in Supplementary data online, *Table S3*. Non-included patients were older, had higher TnT levels and had more comorbidities compared to the included patients. Of the non-included patients, 10% died and 6% were hospitalized for heart failure compared to 4% and 2%, respectively, of the included patients in the present study.

**Table 1:** Population characteristics

	Total (*n* = 653)	SD	IQR	Missing (*n*)
Age	59	11		0
Women	140 (21%)			0
Symptom duration (min)	170		126–271	34
Peak troponin T (ng/L)	2830		1140–5790	0
Infarct size (%)	16		8–25	32
Myocardium at risk (%)	33	11		41
Left ventricular mass (g)	128		109–151	8
Left ventricular ejection fraction (%)	49	10		0
Mean arterial pressure (mmHg)	101	19		0
Risk factors				
Body mass index	27		24–29	0
Diabetes	51 (8%)			0
Active smoker	362 (55%)			1
Past smoker	166 (25%)			1
Hypertension	223 (34%)			1
Hyperlipidaemia	219 (34%)			0
Previous myocardial infarction	25 (4%)			0
Stroke	23 (4%)			0
Congestive heart failure	98 (15%)			0
Creatinine (µmol/L)	75 ± 18			3
Angiography				
TIMI 0–1	401 (61%)			0
TIMI 2–3	252 (39%)			0
LAD culprit	245 (38%)			0
RCA culprit	282 (43%)			0
LCx culprit	50 (8%)			0
Other or indeterminate culprit	76 (12%)			0
Treatment				
Betablocker	603 (92%)			0
ACE-inhibitor	229 (35%)			1
Angiotensin receptor blocker	40 (6%)			3
Calcium channel blocker	51 (8%)			0
Spironolacton	12 (2%)			1
Statin	651 (>99%)			0
Outcome				
Death	26 (4%)			0
Cardiac death	12 (2%)			0
Heart failure	16 (2%)			0

Continuous variables reported by mean (SD = standard deviation) or median [IQR = interquartile range] and categorical variables by absolute value (percentage of population).

ACE, Angiotensin converting enzyme; LAD, left anterior descending artery; LCx, left circumflex artery; RCA, right coronary artery.

### Primary outcome

**Figure 3:**
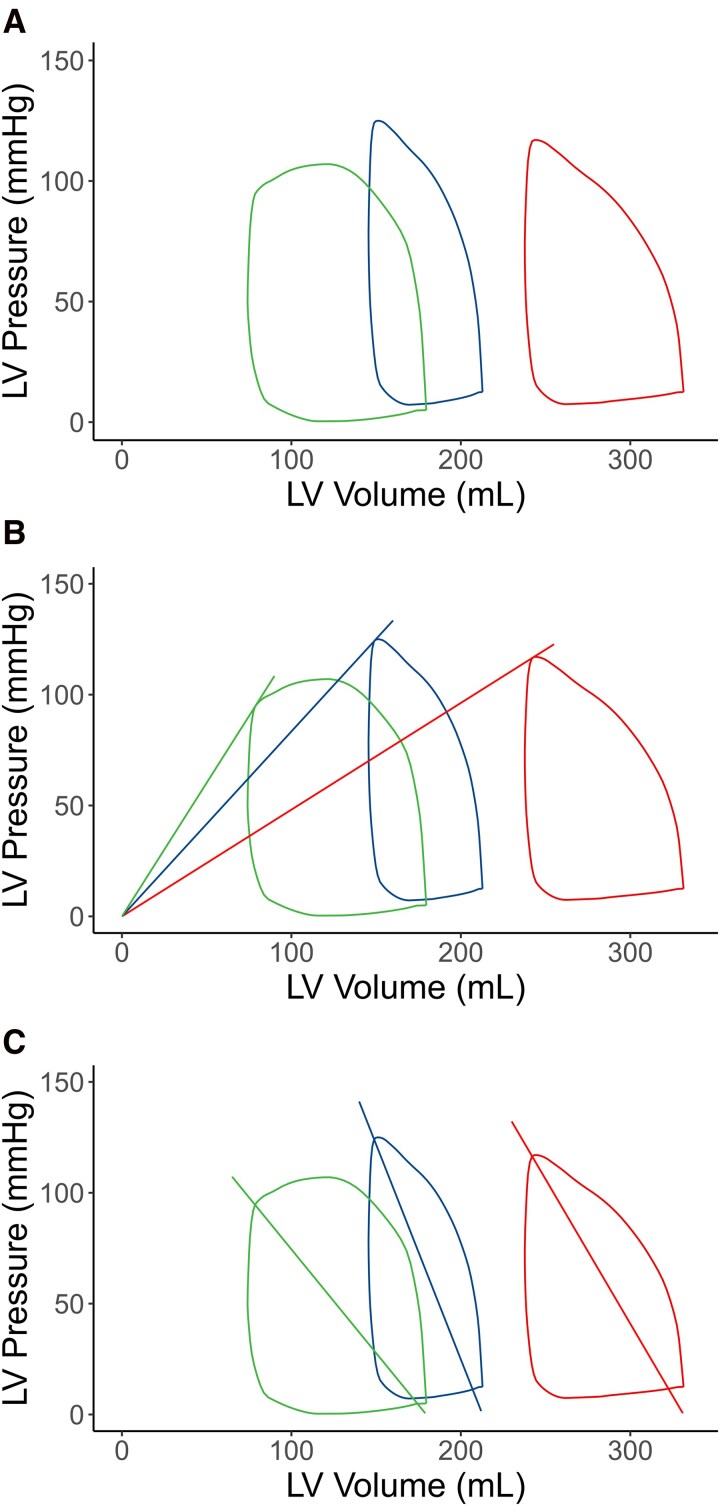
Examples of PV-loops in patients with different outcomes. Examples of non-invasive PV-loops in patients suffering all-cause mortality (red), hospitalization for heart failure (blue) and no adverse outcome (green) (*A*). Lines for contractility and arterial elastance are shown for each patient in (*B*) and (*C*) respectively. Note that contractility was depressed for all-cause mortality and hospitalization for heart failure compared to the patient with no adverse outcome. Also note that arterial elastance was highest in the patient with hospitalization for heart failure.

**Figure 4:**
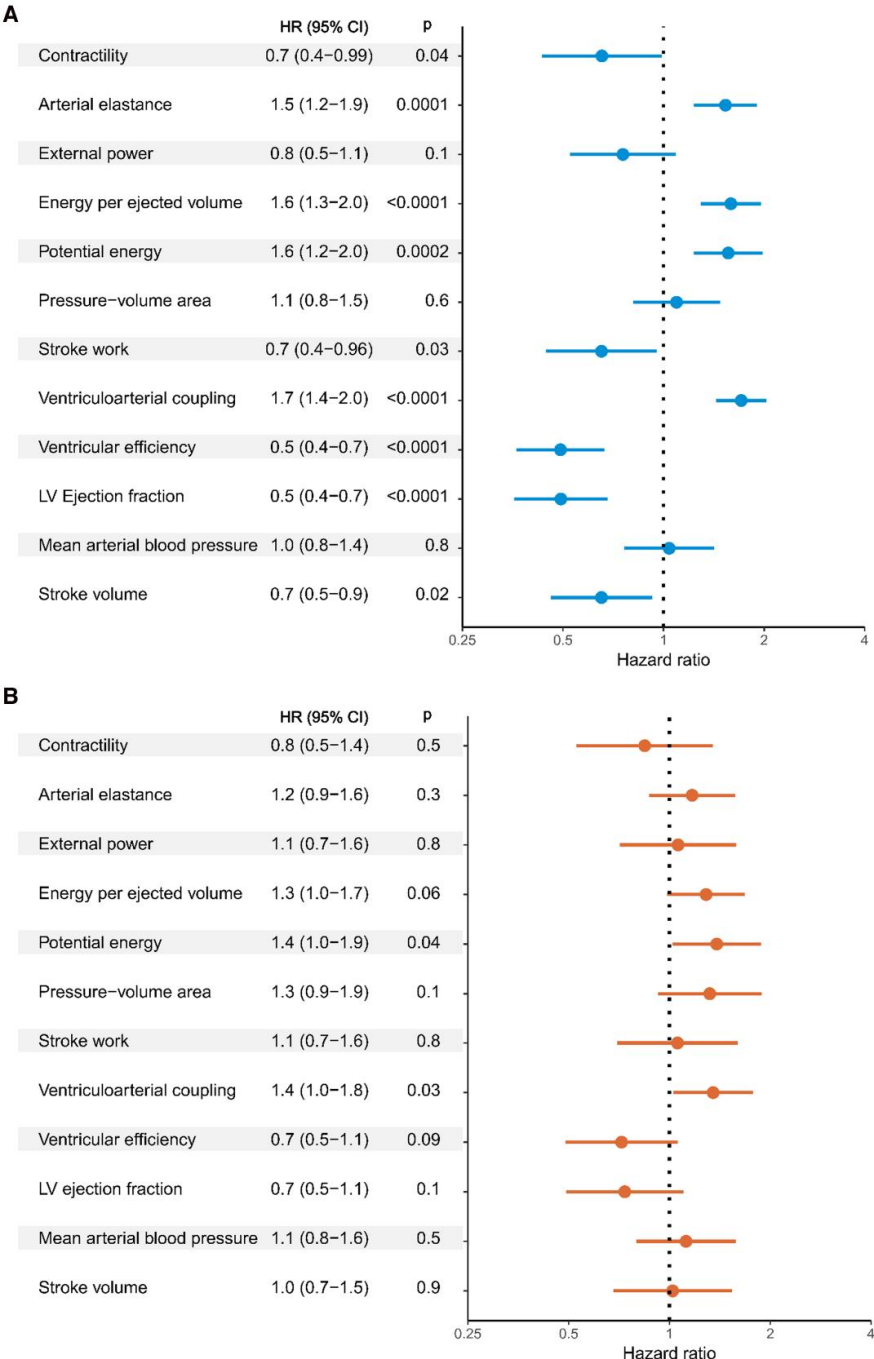
Regression analyses of PV-loop variables for all-cause mortality and heart failure hospitalizations. Unadjusted and adjusted regression models are presented in (*A*) and (*B*) respectively. Each PV-loop variable was tested for separately and adjustments were made for age, sex, and infarct size. LV, left ventricular; HR, hazard ratio.

Examples of PV-loops in patients suffering all-cause mortality, hospitalization for heart failure, and no adverse outcomes, respectively, are shown in *Figure 3*. There were 39 primary outcome events during a median follow-up time of 3.2 (IQR 2.6–4.0) years in the population. The cumulative incidence was 9.7 (CI 5.8–13.6) % for the maximal follow-up time of 4.7 years. Unadjusted cox regression analyses of the primary outcome are shown in *Table 2* and *Figure 4A*. A lower contractility and higher arterial elastance were associated with the primary outcome (HR 0.65, CI 0.43–0.99, and HR 1.53, CI 1.23–1.90, respectively), in accordance with higher ventriculoarterial coupling also being significantly associated with the outcome (HR 1.71, CI 1.43–2.03). A lower stroke work was associated with the primary outcome (HR 0.65, CI 0.45–0.96), however, the association did not remain significant when taking heart rate into account as seen by external power (HR 0.76, CI 0.53–1.09). Cardiac energetics measured by a higher energy per ejected volume (HR 1.59, CI 1.29–1.96) and potential energy (HR 1.56, CI 1.23–1.98), and lower ventricular efficiency (HR 0.49, CI 0.36–0.67) were also associated with the primary outcome. Amongst the conventional measurements, lower LVEF and SV were associated with the primary outcome (HR 0.49, CI 0.36–0.68; and 0.65, 0.46–0.92). Adjusted cox regression analyses of the primary outcome are shown in *Table 3* and *Figure 4B*. All twelve models and all covariates are presented in Supplementary data online, *Table S3*. Due to missing infarct size measurements, *n* = 621 patients with *n* = 34 primary outcome events were used in the adjusted models. Missing infarct size was due to LGE not performed (*n* = 13), poor image quality or technical issues (*n* = 12), and unknown reasons (*n* = 7). Higher potential energy or ventriculoarterial coupling were significantly associated with the primary outcome after confounder adjustments, whereas LVEF, MAP, and SV were not (*Table 3*). The results were unchanged after adding history of diabetes as covariate (see Supplementary data online, *Table S4*). Similar C-indexes were seen between PV-loop variables, conventional measurements, and one model with age, sex and IS as seen in *Table 3*.

**Table 2:** Unadjusted Cox- and Fine and Grey models for primary and secondary outcomes

Outcome	Variable		Unadjusted analysis (*n* = 653)		
			HR (95% CI)	*P*	C-index
Primary outcome (All-cause mortality and hospitalization for heart failure)	PV-loop variables	Contractility	0.65 (0.43–0.99)	0.044	0.63
		Arterial elastance	1.53 (1.23–1.90)	0.00012	0.61
		External power	0.76 (0.53–1.09)	0.13	0.58
		Energy per ejected volume (mJ/mL)	1.59 (1.29–1.96)	<0.0001	0.66
		Potential energy	1.56 (1.23–1.98)	0.00024	0.67
		Pressure–volume area	1.09 (0.81–1.48)	0.56	0.53
		Stroke work	0.65 (0.45–0.96)	0.028	0.62
		Ventriculoarterial coupling	1.71 (1.43–2.03)	<0.0001	0.70
		Ventricular efficiency	0.49 (0.36–0.67)	<0.0001	0.70
	Conventional measurements	LVEF	0.49 (0.36–0.68)	<0.0001	0.70
		MAP	1.04 (0.76–1.42)	0.81	0.51
		SV	0.65 (0.46–0.92)	0.017	0.60
Secondary outcome (All-cause mortality)	PV-loop variables	Contractility	0.47 (0.26–0.83)	0.010	0.65
		Arterial elastance	1.09 (0.76–1.55)	0.64	0.51
		External power	0.66 (0.42–1.05)	0.082	0.58
		Energy per ejected volume (mJ/mL)	1.19 (0.87–1.64)	0.28	0.57
		Potential energy	1.50 (1.11–2.03)	0.0082	0.64
		Pressure–volume area	1.04 (0.71–1.52)	0.84	0.53
		Stroke work	0.64 (0.40–1.02)	0.063	0.59
		Ventriculoarterial coupling	1.40 (1.15–1.72)	0.0011	0.65
		Ventricular efficiency	0.54 (0.37–0.77)	0.00061	0.67
	Conventional measurements	LVEF	0.55 (0.38–0.81)	0.0022	0.66
		MAP	0.77 (0.51–1.16)	0.21	0.57
		SV	0.83 (0.55–1.24)	0.36	0.51
Secondary outcome (Hospitalization for heart failure)	PV-loop variables	Contractility	0.81 (0.35–1.88)	0.63	—
		Arterial elastance	1.98 (1.62–2.42)	<0.0001	—
		External power	0.71 (0.34–1.51)	0.38	—
		Energy per ejected volume (mJ/mL)	1.98 (1.67–2.36)	<0.0001	—
		Potential energy	1.6 (1.21–2.12)	0.001	—
		Pressure–volume area	1.01 (0.64–1.59)	0.98	—
		Stroke work	0.49 (0.20–1.24)	0.13	—
		Ventriculoarterial coupling	2.01 (1.7–2.38)	<0.0001	—
		Ventricular efficiency	0.37 (0.20–0.69)	0.0016	—
	Conventional measurements	LVEF	0.35 (0.17–0.71)	0.0036	—
		MAP	1.33 (0.94–1.88)	0.1	—
		SV	0.35 (0.17–0.73)	0.0053	—

The primary outcome was the composite of all-cause mortality or hospitalization for heart failure. Each variable was standardized and expressed as difference from mean per standard deviation.

CI, confidence interval; HR, hazard ratio; LVEF, left ventricular ejection fraction; MAP, mean arterial blood pressure; SV, stroke volume.

**Table 3:** Adjusted Cox models for the primary outcome

Outcome	Variable		Adjusted analysis (*n* = 621)		
			HR (95% CI)	*P*	C-index
Primary outcome (All-cause mortality and hospitalization for heart failure)	PV-loop variables	Contractility	0.84 (0.53–1.35)	0.48	0.78
		Arterial elastance	1.17 (0.87–1.57)	0.30	0.78
		External power	1.06 (0.71–1.58)	0.77	0.78
		Energy per ejected volume (mJ/mL)	1.29 (0.98–1.68)	0.065	0.79
		Potential energy	1.38 (1.02–1.88)	0.037	0.79
		Pressure–volume area	1.32 (0.92–1.89)	0.13	0.79
		Stroke work	1.06 (0.70–1.60)	0.79	0.78
		Ventriculoarterial coupling	1.35 (1.03–1.78)	0.032	0.78
		Ventricular efficiency	0.72 (0.49–1.06)	0.094	0.79
	Conventional measurements	LVEF	0.74 (0.49–1.10)	0.14	0.79
		MAP	1.12 (0.80–1.58)	0.51	0.78
		SV	1.02 (0.68–1.54)	0.92	0.78
	Confounders	Age, sex and infarct size	—	—	0.78

The primary outcome was the composite of all-cause mortality or hospitalization for heart failure. Each variable was standardized and expressed as difference from mean per standard deviation. Each analysis was adjusted for age, sex, and infarct size.

CI, confidence interval; HR, hazard ratio; LVEF, left ventricular ejection fraction; MAP, mean arterial blood pressure; SV, stroke volume.

### All-cause mortality

The cumulative incidence of all-cause mortality was 5.8 (CI 3.3–8.2) % with 26 deaths during the maximal follow-up time. Contractility, LVEF, potential energy, ventriculoarterial coupling, and ventricular efficiency were significantly associated with all-cause mortality in the unadjusted (*Table 2*) and adjusted analyses (*Table 4*). The PV loop differences are also shown in *Figure 3B*, with the lowest contractility and highest potential energy in the patient with all-cause mortality compared to a patient with hospitalization for heart failure and non-event respectively. All twelve models and all covariates are presented in Supplementary data online, *Table S5*. All variables showed similar C-indexes in the adjusted and unadjusted analyses.

**Table 4:** Adjusted Cox- and Fine and Gray models for secondary outcomes

Outcome	Variable		Adjusted analysis (*n* = 621)		
			HR (95% CI)	*P*	C-index
Secondary outcome (All-cause mortality)	PV-loop variables	Contractility	0.49 (0.26–0.95)	0.035	0.64
		Arterial elastance	0.86 (0.52–1.42)	0.56	0.60
		External power	0.71 (0.43–1.17)	0.18	0.61
		Energy per ejected volume (mJ/mL)	1.10 (0.74–1.63)	0.64	0.60
		Potential energy	1.53 (1.10–2.15)	0.013	0.65
		Pressure–volume area	1.16 (0.78–1.72)	0.47	0.59
		Stroke work	0.78 (0.47–1.29)	0.34	0.59
		Ventriculoarterial coupling	1.36 (1.00–1.84)	0.049	0.63
		Ventricular efficiency	0.52 (0.33–0.83)	0.0056	0.64
	Conventional measurements	LVEF	0.56 (0.34–0.92)	0.021	0.63
		MAP	0.81 (0.52–1.27)	0.37	0.61
		SV	1.01 (0.64–1.59)	0.96	0.59
Secondary outcome (Hospitalization for heart failure)	PV-loop variables	Contractility	1.54 (0.71–3.33)	0.28	—
		Arterial elastance	1.62 (1.27–2.08)	0.00011	—
		External power	1.04 (0.52–2.08)	0.92	—
		Energy per ejected volume (mJ/mL)	1.58 (1.17–2.13)	0.0025	—
		Potential energy	1.15 (0.72–1.83)	0.55	—
		Pressure–volume area	1.04 (0.62–1.74)	0.88	—
		Stroke work	0.91 (0.40–2.06)	0.82	—
		Ventriculoarterial coupling	1.59 (1.11–2.28)	0.011	—
		Ventricular efficiency	0.71 (0.29–1.76)	0.46	—
	Conventional measurements	LVEF	0.68 (0.25–1.84)	0.45	—
		MAP	1.31 (0.93–1.85)	0.12	—
		SV	0.56 (0.29–1.10)	0.092	—

The secondary outcomes were all-cause mortality and hospitalization for heart failure. Each variable was standardized and expressed as difference from mean per standard deviation. Each analysis was adjusted for infarct size.

CI, confidence interval; HR, hazard ratio; LVEF, left ventricular ejection fraction; MAP, mean arterial blood pressure; SV, stroke volume.

### Heart failure

A total of 16 hospitalizations for heart failure, with a cumulative incidence of 4.7 (CI 1.5–7.9) % were seen in the population after adjusting for mortality as a competing risk. Note that proportional model assumptions could not be reliably tested due to the low number of events. Arterial elastance, energy per ejected volume, potential energy, ventriculoarterial coupling, ventricular efficiency, LVEF and SV were associated with heart failure hospitalizations in the unadjusted model. Arterial elastance (HR 1.62, CI 1.27–2.08), energy per ejected volume (HR 1.58, CI 1.17–2.13), and ventriculoarterial coupling (HR 1.59, CI 1.11–2.28) were associated with the outcome after adjustment for infarct size (*Table 4*). This is also shown in *Figure 3C* by the highest arterial elastance in the patient with hospitalization for heart failure compared to a patient with all-cause mortality and non-event respectively. All twelve models and all covariates are presented in Supplementary data online, *Table S6*. Notably, LVEF, MAP, and SV were not significantly associated with hospitalization for heart failure after confounder adjustments.

## Discussion

To the best of our knowledge, this study is first to investigate the prognostic value for clinical outcome of non-invasive PV-loop variables measured during admission in the general STEMI population treated with primary PCI. Non-invasive pressure–volume loop variables are prognostic of all-cause mortality and hospitalizations for heart failure independent of age, sex, and infarct size and may provide incremental prognostic information to left ventricular ejection fraction and infarct size for clinical outcome after myocardial infarction. PV-loop variables might therefore provide a new diagnostic tool for early guiding of treatments aiming to improve LV volumetry or pressure in post-STEMI patients. The study population in the present study has comparable infarct size of 16 [IQR 8–25] %, LVEF of 49 (10) % and time-to-treatment of 170 [IQR 126–271] min to previous CMR outcome studies in optimally treated STEMI populations.^22,25,26^

### PV-loops by CMR

The present study evaluates the prognostic value of non-invasive PV-loops by CMR, showing associations between contractility, arterial elastance, energy per ejected volume, potential energy, stroke work, ventriculoarterial coupling, and ventricular efficiency with all-cause mortality or hospitalization for heart failure. These findings concur with a previous study where non-invasive PV-loop variables agreed with proven prognostic variables such as infarct size measured by CMR immediately after primary PCI.^10^ Furthermore, PV-loop variables have been shown to have predictive value for adverse remodelling.^13^ In that study, however, higher contractility and lower ventricular efficiency were predictive of adverse cardiac remodelling in contrast to the current study showing that lower contractility is associated with increased all-cause mortality. Lower ventricular efficiency has also been associated with major adverse cardiovascular events in a population with heart failure and reduced LVEF, further supporting the prognostic value of non-invasive PV-loops.^14^ Interestingly, potential energy and ventriculoarterial coupling but not LVEF were associated with the primary outcome when adjusting for confounders, suggesting that potential energy and ventriculoarterial coupling could have incremental value when selecting patients for preventive treatment such as primary preventive ICD for counteracting adverse outcomes. A future pathophysiological approach including non-invasive PV-loop variables in the early diagnostic assessment for selecting patients at higher risk needs to be investigated.

### All-cause mortality and heart failure hospitalizations

Higher potential energy and ventriculoarterial coupling, and lower contractility and ventricular efficiency were associated with all-cause mortality in the current study. The relationships between these variables and all-cause mortality have previously not been studied in a revascularized STEMI population. The combination of a low contractility and a high potential energy is seen when the PV-loop is shifted to the right, as seen during ventricular dilatation and adverse remodelling. The combination of a high ventriculoarterial coupling and low ventricular efficiency is seen in a narrow PV-loop, as could be seen with stunned myocardium. Another novel finding in this study is that patients suffering from hospitalization for heart failure were associated with acutely measured arterial elastance, energy per ejected volume and ventriculoarterial coupling were independent of infarct size. Higher values of these variables are seen when end-systolic volume increases at the expense of SV together with a high end-systolic pressure, causing a narrowed width and an increased height of the PV-loop. New treatments aiming to reduce energy consumption in the post-STEMI heart could potentially be useful in the long-term context by lowering the risk of heart failure hospitalizations and possibly all-cause mortality. A treatment aim for reducing cardiac energy consumption could potentially be to decrease ventriculoarterial coupling in the post-STEMI period. It may be that these interventions should focus on decreasing arterial elastance instead of increasing contractility to mitigate the higher mortality risk seen in previous studies.^27^

### Left ventricular ejection fraction, stroke volume, and clinical outcome

There was no significant association between LVEF and the combined outcome of all-cause mortality and hospitalization for heart failure when adjusting for infarct size. This finding is novel as previous studies have investigated the association with a different outcome or have not applied both LVEF and infarct size in the same adjusted model.^22^ The lack of association with LVEF was not explained by collinearity. It could, however, be explained by the confounding effect of infarct size, as the latter is associated with systolic dysfunction as well as mortality or heart failure. The lack of association with LVEF could be due to underpowering by the low number of events seen in the adjusted model, as missing infarct size led to five primary outcome events being omitted from the analysis. The lack of association could also be explained by the sample selection as healthier patients with higher LVEF could make it to the CMR investigations. The mean (SD) LVEF of 49 (10)% in the current study is, however, comparable to previous post-STEMI CMR studies with examinations performed acutely after revascularization.^28,29^ Despite the non-significant association between LVEF and the primary outcome, similar concordance indexes between LVEF and PV-loop variables in the current study indicate a similar ability to discriminate between future clinical outcomes. Stroke volume was neither associated with the secondary outcomes of all-cause mortality nor heart failure hospitalizations after adjustments for infarct size. Stroke volume-derived PV loop variables such as ventricular efficiency, arterial elastance, and energy per ejected volume were, however, associated with secondary outcomes. This discrepancy could be explained by the end-systolic pressures and absolute end-systolic and/or -diastolic volumes needed to calculate these variables. The residual volume together with the intraventricular pressure, rather than stroke volume alone, seem to contribute to the association with secondary outcomes. Thus, the combined effects of intraventricular pressures and -volumes seen in the PV-loop variables seem to offer more prognostic information compared to stroke volume alone.

### Clinical applications for PV loop variables

The results from this and a previous study suggest that non-invasive PV loop variables by CMR could be used early in assessing the risk of developing adverse outcomes.^13^ Additionally, potential energy and ventriculoarterial coupling showed relevance for the risk of adverse outcomes and present potential therapeutic goals that should be evaluated in future clinical trials. The incremental value to LVEF and infarct size could be of future use when designing non-contrast CMR protocols for STEMI patients.

### Limitations

The present study has a selection bias for those who underwent CMR compared to those who did not. In the patient group not included in the analysis, 10% died and 6% were hospitalized for heart failure compared to 4% who died and 2% that were hospitalized for heart failure of the included patients in the present study. The selection bias and the optimal treatment of this STEMI population with primary PCI likely explain the few adverse outcomes in the population. A selected patient population can be beneficial when showing proof-of-concept early in the development of a new method. The low number of outcome events seen in the study is a limitation for the non-statistically significant variables. Adjustments could not be made for additional risk factors due to the limited number of events in the population. Infarct size has previously been shown to be independently associated with the primary outcome, however, and the inclusion of this variable in the adjusted models should be considered a strength in the study. The infarct size was measured within 48 h of the insult, which is earlier than guidelines recommend and could lead to an overestimation of the infarct size due to acute oedema.^30^ Since the timepoint of imaging was similar for all study subjects this overestimation should, however, be similar between different patients. Myocardium at risk and infarct size were assessed by the 2- and 5 SD from remote methods, which have been shown to be sub-optimal for quantifying myocardial extents of infarct or oedema.^31^ Considering this is a single-centre, single-vendor study in a controlled setting the 2- and 5 SD method should still provide acceptably low bias. Myocardial haemorrhage has been shown to be an independent prognostic marker after STEMI, but was not adjusted for in the present study, which is a limitation.

## Conclusion

Non-invasive pressure–volume loop variables are prognostic of all-cause mortality and hospitalizations for heart failure independent of age, sex, and infarct size and may provide incremental prognostic information to left ventricular ejection fraction and infarct size for clinical outcome after myocardial infarction. Thus, non-invasive PV-loop variables could potentially be used for early treatment guidance in post-STEMI patients.

## Supplementary Material

qyag055_Supplementary_Data

## Lead author biography

This study was a collaboration between Lund University and Copenhagen University. Theodor Lav is a PhD student in the Cardiac MR Group at the Department of Clinical Physiology, Lund University, and working as a physician completing the foundation years at Skåne University Hospital, Malmö, Sweden. During the course of this study, he was a visiting PhD student at University of Copenhagen.
