# Supplementary material for: Non-invasive pressure–volume loops by cardiovascular magnetic resonance and outcome in ST–elevation myocardial infarction
Source: Eur Heart J Imaging Methods Pract. 2026 Mar 29;4(1):qyag055. doi: 10.1093/ehjimp/qyag055 (PMC13100501; doi:10.1093/ehjimp/qyag055)

**Supplementary materials**

**Supplementary Table 1.** Formulas for PV-loop variables and conventional measurements. DBP=diastolic blood pressure. EDV=End-diastolic volume. ESV=End-systolic volume. LVEF=left ventricular ejection fraction. MAP=mean arterial blood pressure. SBP=systolic blood pressure. SV=stroke volume.

|  | **Variable** | **Formula** | **Unit** |
| --- | --- | --- | --- |
| **PV-loop variables** | External power | $Stroke work\cdot Heart rate$ | J/s |
|  | Energy per ejected volume | $\frac{Stroke work+Potential energy}{Stroke volume}$ | mJ/mL |
|  | Ventricular efficiency | $\frac{Stroke work}{Stroke work+Potential energy}$ | % |
|  | PV loop area | $Stroke work+Potential energy$ | J |
|  | Ventriculoarterial coupling | $\frac{Arterial elastance}{\mathrm{Contractility}}$ | - |
| **Conventional measurements** | LVEF | $\frac{\mathrm{SV}}{\mathrm{EDV}}$ | % |
|  | MAP | $\frac{SBP-DBP}{3}+DBP$ | mmHg |
|  | SV | $EDV-ESV$ | mL |

**Supplementary Table 2.** Median, interquartile range, mean and standard deviation for PV-loop variables.

| PV-loop variable | Median [Interquartile range] | Mean | Standard deviation |
| --- | --- | --- | --- |
| Contractility (mmHg/mL) | 1.3 [1.0 – 1.6] | 1.4 | 0.5 |
| Ea (mmHg/mL) | 1.5 [1.2 – 1.9] | 1.6 | 0.6 |
| EP (J/s) | 1.4 [1.1 – 1.7] | 1.4 | 0.5 |
| EpV (mJ/mL) | 21 [18 – 25] | 22 | 6 |
| PE (J) | 0.7 [0.5 – 0.9] | 0.7 | 0.3 |
| PVA (J) | 1.8 [1.5 – 2.2] | 1.9 | 0.5 |
| SW (J) | 1.1 [0.9 – 1.3] | 1.2 | 0.4 |
| VAC | 1.1 [0.9 – 1.5] | 1.3 | 0.6 |
| VE (%) | 63 [54 – 71] | 62 | 12 |
| SV | 85 [71 – 99] | 86 | 21 |
| LVEF | 50 [42 – 56] | 49 | 10 |
| IS | 16 [8 – 25] | 17 | 12 |
| MAP | 100 [89 – 111] | 101 | 19 |

**Supplementary Table 3. Patient characteristics in non-included and included patients from the study.**

|  | **Included patients**  **(n=653)** | **SD [IQR]** | **Missing**  **(n)** | **Non-included patients (n=967)** | **SD [IQR]** | **Missing (n)** | **p** |
| --- | --- | --- | --- | --- | --- | --- | --- |
| Age (years) | 59 | 11 | 0 | 64 | 12 | 0 | <0.0001 |
| Symptom duration (min) | 170 | [126-271] | 34 | 185 | [136-285] | 31 | 0.012 |
| Peak Troponin T (ng/L) | 2830 | [1140-5790] | 0 | 3240 | [1160-6740] | 5 | <0.0001 |
| **Comorbidities** | | | | | | | |
| - Diabetes | 51 (8%) |  | 0 | 108 (11%) |  | 0 | 0.032 |
| - Active smoker | 362 (55%) |  | 1 | 476 (49%) |  | 0 | 0.016 |
| - Past smoker | 166 (25%) |  | 1 | 303 (31%) |  | 0 | 0.012 |
| - Hypertension | 223 (34%) |  | 1 | 437 (45%) |  | 0 | <0.0001 |
| - Hyperlipidemia | 219 (34%) |  | 0 | 362 (37%) |  | 0 | 0.12 |
| - Previous AMI | 25 (4%) |  | 0 | 79 (8%) |  | 0 | 0.00069 |
| - Previous stroke | 23 (4%) |  | 0 | 50 (5%) |  | 0 | 0.15 |
| - Chronic heart failure | 98 (15%) |  | 0 | 189 (20%) |  | 1 | 0.022 |
| - Creatinine (µmol/L) | 75 | 18 | 3 | 81 | 29 | 4 | <0.0001 |
| **Medications** | | | | | | | |
| - Betablocker | 603 (92%) |  | 0 | 858 (90%) |  | 13 | 0.12 |
| - ACE-inhibitor | 229 (35%) |  | 1 | 340 (36%) |  | 14 | 0.86 |
| - ARB | 40 (6%) |  | 3 | 89 (9%) |  | 13 | 0.028 |
| - CCB | 51 (8%) |  | 0 | 89 (9%) |  | 13 | 0.33 |
| - Spironolacton | 12 (2%) |  | 1 | 40 (4%) |  | 14 | 0.013 |
| - Statin | 651 (>99%) |  | 0 | 925 (97%) |  | 15 | 0.00039 |
| **Angiography** | | | | | | | |
| - TIMI 0-1 | 401 (61%) |  | 0 | 577 (60%) |  | 0 | 0.52 |
| - TIMI 2-3 | 252 (39%) |  | 0 | 390 (40%) |  | 0 | 0.52 |
| - LAD culprit | 245 (38%) |  | 0 | 378 (39%) |  | 0 | 0.56 |
| - RCA culprit | 282 (43%) |  | 0 | 368 (38%) |  | 0 | 0.044 |
| - LCx culprit | 50 (8%) |  | 0 | 107 (11%) |  | 0 | 0.029 |
| - Other | 76 (12%) |  | 0 | 114 (12%) |  | 0 | 0.99 |
| **Outcome** | | | | | | | |
| - Death | 26 (4%) |  | 0 | 97 (10%) |  | 17 | <0.0001 |
| - Cardiac death | 12 (2%) |  | 0 | 54 (6%) |  | 17 | 0.00023 |
| - Heart failure | 16 (2%) |  | 0 | 54 (6%) |  | 17 | 0.0028 |

**Supplementary Table 4. Multivariate regression analyses of PV-loop variables for all-cause mortality and heart failure hospitalizations.** All twelve models with all covariates are presented below. CI=confidence interval. HR=Hazard ratio.

| **Model** | **Variable** | **HR** | **Lower CI** | **Upper CI** | **p** |
| --- | --- | --- | --- | --- | --- |
| 1 | Stroke volume | 1.022302 | 0.678916 | 1.539369 | 0.915886 |
|  | Age | 1.07954 | 1.04157 | 1.118894 | 2.8E-05 |
|  | Sex | 1.912106 | 0.704678 | 5.188396 | 0.203115 |
|  | Infarct size | 1.040835 | 1.011687 | 1.070824 | 0.00575 |
| 2 | Mean arterial blood pressure | 1.120794 | 0.796376 | 1.577371 | 0.513068 |
|  | Age | 1.079773 | 1.042885 | 1.117965 | 1.51E-05 |
|  | Sex | 1.891161 | 0.717233 | 4.986513 | 0.197711 |
|  | Infarct size | 1.038748 | 1.011415 | 1.06682 | 0.005202 |
| 3 | Ejection fraction | 0.7353 | 0.490985 | 1.101185 | 0.135648 |
|  | Age | 1.075522 | 1.038633 | 1.113721 | 4.34E-05 |
|  | Sex | 1.795569 | 0.679831 | 4.742456 | 0.237527 |
|  | Infarct size | 1.024482 | 0.992022 | 1.058004 | 0.140927 |
| 4 | Ventricular efficiency | 0.71941 | 0.489509 | 1.057286 | 0.093659 |
|  | Age | 1.074299 | 1.037388 | 1.112523 | 5.88E-05 |
|  | Sex | 1.776169 | 0.672452 | 4.691451 | 0.246372 |
|  | Infarct size | 1.022873 | 0.990741 | 1.056048 | 0.164908 |
| 5 | Ventriculoarterial coupling | 1.350239 | 1.026184 | 1.776627 | 0.031988 |
|  | Age | 1.073355 | 1.036241 | 1.111797 | 8.05E-05 |
|  | Sex | 1.769905 | 0.669025 | 4.682279 | 0.250057 |
|  | Infarct size | 1.021446 | 0.989888 | 1.054009 | 0.185097 |
| 6 | Stroke work | 1.057174 | 0.697519 | 1.602275 | 0.793273 |
|  | Age | 1.080188 | 1.042345 | 1.119405 | 2.24E-05 |
|  | Sex | 1.887161 | 0.702956 | 5.066285 | 0.207513 |
|  | Infarct size | 1.041671 | 1.012572 | 1.071605 | 0.004739 |
| 7 | Pressure-volume area | 1.319166 | 0.922932 | 1.88551 | 0.128534 |
|  | Age | 1.083673 | 1.046259 | 1.122424 | 7.38E-06 |
|  | Sex | 1.576787 | 0.577204 | 4.307414 | 0.374459 |
|  | Infarct size | 1.038153 | 1.011041 | 1.065991 | 0.00555 |
| 8 | Potential energy | 1.38419 | 1.020541 | 1.877419 | 0.036553 |
|  | Age | 1.078653 | 1.042104 | 1.116484 | 1.67E-05 |
|  | Sex | 1.563588 | 0.581557 | 4.203898 | 0.375731 |
|  | Infarct size | 1.025509 | 0.995933 | 1.055963 | 0.091601 |
| 9 | Energy per ejected volume | 1.286506 | 0.98476 | 1.680711 | 0.064696 |
|  | Age | 1.075669 | 1.03865 | 1.114008 | 4.46E-05 |
|  | Sex | 1.843188 | 0.697976 | 4.867418 | 0.217121 |
|  | Infarct size | 1.027623 | 0.997592 | 1.058558 | 0.071762 |
| 10 | External power | 1.061197 | 0.711686 | 1.582355 | 0.770748 |
|  | Age | 1.080561 | 1.04225 | 1.12028 | 2.59E-05 |
|  | Sex | 1.895443 | 0.711964 | 5.046187 | 0.200562 |
|  | Infarct size | 1.04105 | 1.01343 | 1.069423 | 0.003364 |
| 11 | Arterial elastance | 1.168935 | 0.86828 | 1.573696 | 0.303511 |
|  | Age | 1.075435 | 1.037976 | 1.114246 | 5.81E-05 |
|  | Sex | 2.081351 | 0.780042 | 5.553579 | 0.143227 |
|  | Infarct size | 1.034406 | 1.005257 | 1.0644 | 0.020367 |
| 12 | Contractility | 0.843744 | 0.527506 | 1.349566 | 0.478324 |
|  | Age | 1.080197 | 1.04322 | 1.118485 | 1.42E-05 |
|  | Sex | 1.757852 | 0.648909 | 4.761908 | 0.267248 |
|  | Infarct size | 1.035275 | 1.005721 | 1.065696 | 0.018973 |

**Supplementary Table 5.** Adjusted analysis (n=621) for the composite of all-cause mortality or hospitalization for heart failure as outcome. Each variable was adjusted for age, sex, infarct size, and diabetes. CI=confidence interval. LVEF=Left ventricular ejection fraction. HR=Hazard ratio. SV=Stroke volume.

| **Variables** | **HR (95% CI)** | **p** | **C-index** |
| --- | --- | --- | --- |
| Contractility | 0.73 (0.3-1.7) | 0.5 | 0.78 |
| Arterial elastance | 1.3 (0.79-2.2) | 0.3 | 0.78 |
| External power | 1.1 (0.47-2.7) | 0.8 | 0.78 |
| Energy per ejected volume (mJ/mL) | 1.0 (0.998-1.1) | 0.06 | 0.78 |
| Potential energy | 2.8 (1.1-7.2) | 0.03 | 0.79 |
| Pressure-volume area | 1.7 (0.86-3.2) | 0.1 | 0.78 |
| Stroke work | 1.1 (0.40-3.2) | 0.8 | 0.78 |
| Ventriculoarterial coupling | 1.6 (1.1-2.6) | 0.03 | 0.78 |
| Ventricular efficiency | 0.97 (0.94-1.0) | 0.09 | 0.79 |
| LVEF | 0.97 (0.93-1.0) | 0.1 | 0.78 |
| MAP | 1.0 (0.99-1.0) | 0.5 | 0.78 |
| SV | 1.0 (0.98-1.0) | 0.9 | 0.78 |

**Supplementary Table 6. Multivariate regression analyses of PV-loop variables for all-cause mortality.** All twelve models with all covariates are presented below. CI=confidence interval. HR=Hazard ratio.

| **Model** | **Variable** | **HR** | **Lower CI** | **Upper CI** | **p** |
| --- | --- | --- | --- | --- | --- |
| 1 | Stroke volume | 1.011213 | 0.641538 | 1.593907 | 0.961693 |
|  | Infarct size | 1.01807 | 0.981961 | 1.055507 | 0.331067 |
| 2 | Stroke work | 0.781206 | 0.473056 | 1.290086 | 0.334666 |
|  | Infarct size | 1.011915 | 0.977228 | 1.047834 | 0.505687 |
| 3 | Ejection fraction | 0.56168 | 0.344513 | 0.915741 | 0.020728 |
|  | Infarct size | 0.991987 | 0.954569 | 1.030873 | 0.681747 |
| 4 | Ventricular efficiency | 0.519928 | 0.327431 | 0.825595 | 0.005566 |
|  | Infarct size | 0.987754 | 0.950872 | 1.026067 | 0.525682 |
| 5 | Ventriculoarterial coupling | 1.358018 | 1.001082 | 1.842221 | 0.049193 |
|  | Infarct size | 0.999439 | 0.960819 | 1.039611 | 0.977735 |
| 6 | Potential energy | 1.53402 | 1.095359 | 2.148354 | 0.012775 |
|  | Infarct size | 1.003065 | 0.968436 | 1.038932 | 0.864457 |
| 7 | Energy per ejected volume | 1.098129 | 0.73855 | 1.632777 | 0.643712 |
|  | Infarct size | 1.014105 | 0.977106 | 1.052505 | 0.460144 |
| 8 | External power | 0.707755 | 0.428869 | 1.167997 | 0.176251 |
|  | Infarct size | 1.013287 | 0.979898 | 1.047813 | 0.440052 |
| 9 | Contractility | 0.494938 | 0.257485 | 0.951374 | 0.034904 |
|  | Infarct size | 1.005312 | 0.971446 | 1.040359 | 0.761863 |
| 10 | Arterial elastance | 0.860646 | 0.522183 | 1.41849 | 0.556086 |
|  | Infarct size | 1.022325 | 0.985481 | 1.060548 | 0.238399 |
| 11 | Pressure-volume area | 1.156 | 0.7774 | 1.718 | 0.474 |
|  | Infarct size | 1.017 | 0.9835 | 1.053 | 0.317 |
| 12 | Mean arterial blood pressure | 0.814694 | 0.522733 | 1.269722 | 0.365352 |
|  | Infarct size | 1.019263 | 0.985635 | 1.054039 | 0.264998 |

**Supplementary Table 7. Multivariate regression analyses of PV-loop variables for hospitalization for heart failure.** All twelve models with all covariates are presented below. CI=confidence interval. HR=Hazard ratio.

| **Model** | **Variable** | **HR** | **Lower CI** | **Upper CI** |
| --- | --- | --- | --- | --- |
| 1 | Stroke volume | 0.563 | 0.289 | 1.10 |
|  | Infarct size | 1.071 | 1.025 | 1.12 |
| 2 | Pressure-volume area | 1.04 | 0.622 | 1.74 |
|  | Infarct size | 1.09 | 1.038 | 1.14 |
| 3 | Ejection fraction | 0.677 | 0.249 | 1.84 |
|  | Infarct size | 1.067 | 1.002 | 1.14 |
| 4 | Ventricular efficiency | 0.713 | 0.289 | 1.76 |
|  | Infarct size | 1.069 | 1.002 | 1.14 |
| 5 | Ventriculoarterial coupling | 1.59 | 1.11 | 2.28 |
|  | Infarct size | 1.06 | 1.00 | 1.11 |
| 6 | Potential energy | 1.15 | 0.722 | 1.83 |
|  | Infarct size | 1.08 | 1.029 | 1.14 |
| 7 | Energy per ejected volume | 1.58 | 1.17 | 2.13 |
|  | Infarct size | 1.06 | 1.01 | 1.12 |
| 8 | External power | 1.04 | 0.517 | 2.08 |
|  | Infarct size | 1.09 | 1.034 | 1.14 |
| 9 | Contractility | 1.54 | 0.708 | 3.33 |
|  | Infarct size | 1.10 | 1.035 | 1.18 |
| 10 | Arterial elastance | 1.62 | 1.27 | 2.08 |
|  | Infarct size | 1.07 | 1.02 | 1.11 |
| 11 | Stroke work | 0.909 | 0.40 | 2.06 |
|  | Infarct size | 1.084 | 1.03 | 1.14 |
| 12 | Mean arterial blood pressure | 1.31 | 0.93 | 1.85 |
|  | Infarct size | 1.09 | 1.04 | 1.14 |

**Supplementary Figure 1. Correlation matrix of age, infarct size, conventional measurements and PV loop variables.** Spearman correlation was applied between each association. Note that ventriculoarterial coupling and ejection fraction follows a multiplicative inverted relationship.


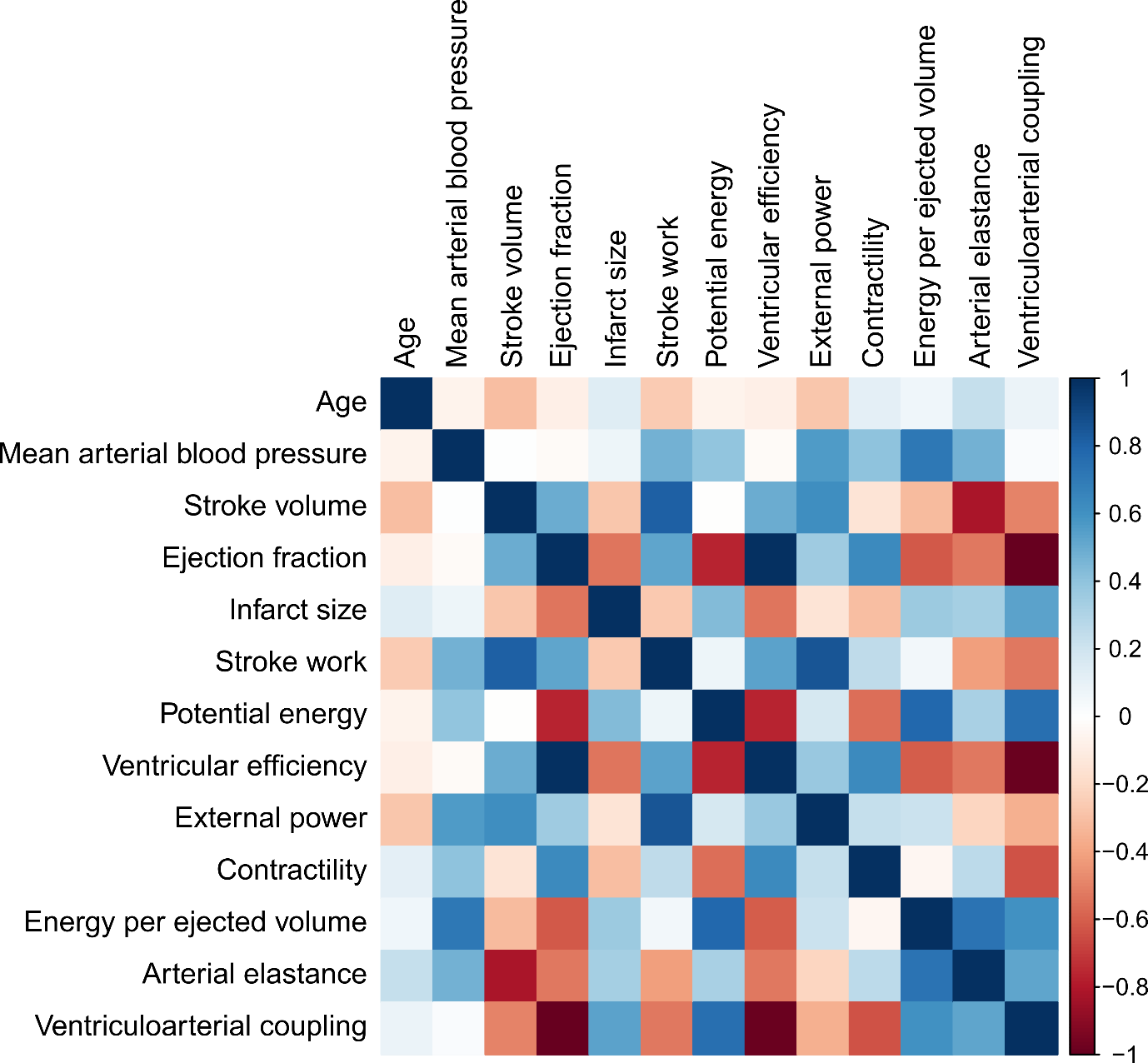

Supplement: qyag055_Supplementary_Data [file qyag055_supplementary_data.docx]
